# Effect of beta‐blockade on mortality in patients with cardiac amyloidosis: A systematic review and meta‐analysis

**DOI:** 10.1002/ehf2.14975

**Published:** 2024-07-23

**Authors:** Chun Shing Kwok, Chern Hsiang Choy, Jennifer Pinney, Jonathan N. Townend, Carol Whelan, Marianna Fontana, Julian D. Gillmore, Richard P. Steeds, William E. Moody

**Affiliations:** ^1^ Department of Cardiology, Queen Elizabeth Hospital Birmingham University Hospitals of Birmingham NHS Foundation Trust Birmingham UK; ^2^ Department of Nephrology, Queen Elizabeth Hospital Birmingham University Hospitals of Birmingham NHS Foundation Trust Birmingham UK; ^3^ Institute of Cardiovascular Sciences, College of Medical and Dental Sciences University of Birmingham Birmingham UK; ^4^ Division of Medicine, National Amyloidosis Centre University College London, Royal Free Hospital London UK

**Keywords:** Beta‐blocker, Cardiac amyloidosis, Heart failure, Light chain amyloid cardiomyopathy, Transthyretin amyloid cardiomyopathy

## Abstract

**Aims:**

The efficacy of beta‐blockers in cardiac amyloidosis (CA) is unclear, and concerns persist that neurohormonal blockade could worsen symptoms of heart failure. We aimed to assess whether beta‐blocker therapy is associated with improved survival in patients with CA.

**Methods and results:**

We conducted a systematic review and meta‐analysis to examine the impact of beta‐blocker therapy on mortality in patients with CA. A search of MEDLINE and EMBASE was performed in August 2023. Data were extracted from observational studies and synthesized with pooling and random effects meta‐analysis. Thirteen studies including 4215 patients with CA were incorporated in this review (3688 transthyretin amyloid cardiomyopathy (ATTR‐CM), 502 light chain amyloid cardiomyopathy (AL‐CM), 25 not specified; age 74.8 ± 5.5 years, 76% male). Over half of the cohort (52%) received beta‐blockers and the rate of beta‐blocker withdrawal was 28%. All‐cause mortality was 33% (range: 13–51%) after a median follow‐up ranging from 13 to 36 months. There was an inverse association between the pooled risk of mortality and the use of beta‐blocker therapy at any time point (RR 0.48, 95% CI 0.29–0.80, *I* ^2^ = 83%, *P* = 0.005, seven studies). There was no association between mortality and beta‐blocker use (RR 0.65, 95% CI 0.29–1.47, *I* ^2^ = 88%, *P* = 0.30) in the three studies that only included patients with ATTR‐CM. The three studies that included patients with both ATTR‐CM and AL demonstrated an association of beta‐blocker use with reduced mortality (OR 0.43, 95% CI 0.29–0.63, *I* ^2^ = 4%, *P* < 0.001). The only study that solely included 53 patients with AL‐CM, demonstrated improved survival among the 53% who were able to tolerate beta‐blocker therapy (RR 0.26, 95% CI 0.08–0.79, *P* = 0.02). The absence of information on staging of CA is an important limitation of this study.

**Conclusions:**

Treatment with beta‐blockers may be associated with a survival benefit in patients with CA, but these findings are subject to selection and survivor biases. Definitive prospective randomized trials of conventional heart failure therapies are needed in CA.

## Introduction

There is increasing realization that cardiac amyloidosis (CA) can no longer be considered a rare disease entity. Over the last decade, there has been a near exponential rise in the number of patients diagnosed with transthyretin amyloid cardiomyopathy (ATTR‐CM). This has been fuelled by the emergence of novel TTR disease‐modifying therapies leading to greater disease awareness, as well as improvements in diagnostic pathways coupled with better access to multimodality imaging.^1^ Wild‐type ATTR (ATTRwt) amyloidosis primarily results in progressive and fatal heart failure from a restrictive cardiomyopathy (ATTRwt‐CM) and accounts for up to 13% of all patients diagnosed with heart failure with preserved ejection fraction (HFpEF).^2^ In the United States, there are now approximately 5000 to 7000 new cases identified annually^3^ and the prevalence among Nordic countries ranges from 1.4 to 5.0 per 100 000 inhabitants.^4^ In contrast, the incidence of light chain amyloidosis (AL amyloidosis) has not changed over decades; in an evaluation of Olmsted County in the United States, there was no significant difference in the incidence rate of 1.2 per 100 000 person‐years from 1990–2015 and the rate accounting for the preceding 30 years.^5^

At the time of diagnosis, more than half of patients with ATTRwt‐CM take beta‐blockers for left ventricular systolic dysfunction, rate control of atrial fibrillation, coronary disease or a history of ventricular tachyarrhythmia.^6^, ^7^, ^8^ None of the randomized controlled trials of heart failure, atrial fibrillation, or ischaemic heart disease, however, have knowingly included patients with CA. There is long‐held concern regarding the potential for beta‐blockers to worsen symptoms in patients with CA.^9^ Beta‐blocker therapy can lead to chronotropic incompetence and an impaired ability to augment cardiac output, resulting from a relatively fixed stroke volume secondary to low ventricular capacitance and altered ventricular–vascular coupling.^10^ This pathophysiological concept is at odds, however, with findings from the largest observational study to date examining the effect of beta‐blockers on mortality in ATTR‐CM, in which low dose beta‐blockade was associated with improved survival in a subset of patients with a left ventricular ejection fraction of <40%.^11^ The evidence is conflicting, however, with an earlier study suggesting that withdrawal of beta‐blockers during follow‐up of patients with ATTR‐CM was associated with improved survival.^12^ Much of the data examining neurohormonal blockade in CA is of limited quality taken from small, retrospective cohort studies.^13^, ^14^, ^15^, ^16^ These are inherently subject to selection bias, which may account for the inconsistent findings and contribute to the lack of consensus between national and international society guidelines on this subject (all level of evidence: C).^17^

To address the urgent need to better understand if there is a role for beta‐blockade in the increasing number of patients being diagnosed with CA, we performed a systematic review and meta‐analysis of observational studies examining the association of beta‐blocker therapy with survival.

## Methods

This systematic review and meta‐analysis was reported in accordance with the Preferred Reporting Items for Systematic Reviews and Meta‐analysis (PRISMA) guidelines (Data S1).^18^

### Eligibility criteria

We included studies that evaluated patients with ATTR and/or AL cardiac amyloidosis examining the association of beta‐blockers with mortality, functional status and tolerability. Tolerability was assessed by examining the frequency of adverse side effects that were reported by studies associated with beta‐blocker use. Only studies that reported numerical events or adjusted risk ratios, odds ratios, and hazard ratios were included. Systematic reviews, case reports, and editorials without original data were excluded.

### Information sources and search strategy

We searched MEDLINE and EMBASE in August 2023 using the OVID platform to identify relevant studies. The exact search terms were (amyloid*) and (beta‐adrenergic antagonist* OR beta‐blocker*).

### Screening, quality assessment, and data extraction

The screening for potentially relevant studies was carried out independently by two reviewers (C.S.K and C.H.C). Full copies of potentially relevant studies were retrieved and reviewed for inclusion. The final decision regarding the inclusion of studies was determined by two senior authors (J.D.G. and W.E.M).

Data were collected on study design, country of origin, year of conduct, number of participants, age of the participants, sex, and participant inclusion criteria. Additional information was collected on amyloid typing, the prevalence of atrial fibrillation or flutter, hypertension, coronary artery disease, the proportion of patients on beta‐blockers, withdrawal of beta‐blocker therapy, dosing, tolerability, adverse side effects, duration of follow up, and mortality.

The quality assessment was undertaken using the Ottawa‐Newcastle score.^19^ Studies were deemed to be representative if they included patients with a confirmed diagnosis of ATTR‐CM or AL‐CM. Other areas assessed included the selection of a non‐exposed cohort, ascertainment of beta‐blocker exposure, demonstration that the safety or mortality outcome was not present at the start of study, use of adjustments to account for confounders, reliability of the outcome assessment, adequacy of the follow up, and the extent of missing data. Studies with follow up in‐hospital or beyond were classified as low risk of bias and studies with >10% missing data were deemed to be of lower quality. A total of nine possible stars were assigned to each study.^19^

### Synthesis

**Figure 1:**
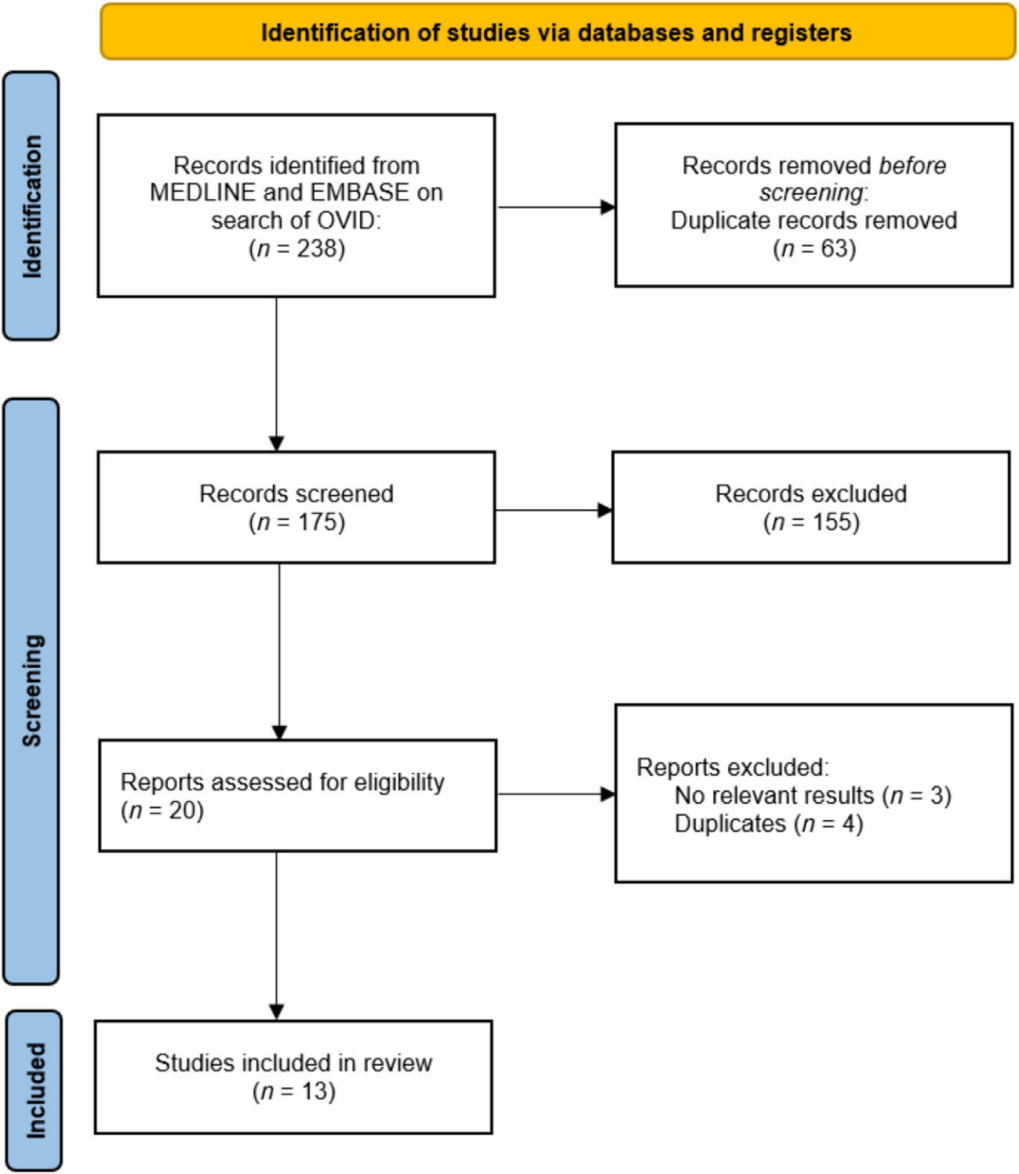
PRISMA flow diagram of study inclusion.

The process of study inclusion is illustrated in the PRISMA flow diagram (*Figure* *1*). We used Review Manager 5.3 (Nordic Cochrane Group, Copenhagen) to perform random effects meta‐analysis using the inverse variance method. Only the most adjusted estimates of risk or hazards were used to determine the risk of mortality with or without beta‐blockers at any time point. The *I* ^2^ statistic was used to estimate statistical heterogeneity where an *I* ^2^ value of 30% to 60% represents a moderate degree of statistical heterogeneity.^20^ We also numerically pooled events according to previously published methods.^21^

## Results

### Study selection

The process of study selection is illustrated in *Figure* *1*. From 238 studies identified in the search, a total of 13 were included in this review.^7^, ^10^, ^11^, ^12^, ^13^, ^14^, ^15^, ^16^, ^22^, ^23^, ^24^, ^25^, ^26^ A summary of the 13 studies that met the inclusion criteria is shown in *Table* *1*. There were three prospective cohort studies and 10 retrospective cohort studies, and these studies took place in the United States, Italy, Spain, Greece, Portugal, and the United Kingdom between 1990 and 2022. A total of 4215 patients with CA were included in the analysis (502 AL‐CM, 3688 ATTR‐CM, 25 not specified); more than half (*n* = 2371) were patients with ATTRwt‐CM included from a UK based study.^12^ The study quality assessment is shown in Supplementary Table S1. A total of four studies included patients with ATTR‐CM only, and two studies exclusively assessed patients with AL‐CM, while the remaining seven studies included a mix. All studies had reliable data on the number of patients on beta‐blockers, ascertainment of exposure, demonstration that mortality or adverse events were not present at the start of study, and ascertainment of outcomes. Statistical adjustment with multivariable regression to account for differences in patient variables was undertaken in seven studies. Follow‐up was available in all but one study^24^ and with the exception of one report,^11^ studies had <10% missing data. In quality assessment, the mean number of stars was 7.3 ± 1.3, and two studies had the maximum nine stars.

**Table 1:** Study design and participant characteristics of included studies

Study ID	Study design; country; year	No. of patients	Mean age (years)	% Male	Inclusion criteria
Aimo 2020	Retrospective cohort study; Italy; 2009 to 2019.	99	Median 80	71.7%	Patients were diagnosed with cardiac amyloidosis at a tertiary centre in Italy.
Austin 2009	Retrospective cohort study; United States; 1998 to 2003.	45	66	‐	Patients were diagnosed with cardiac amyloidosis by endomyocardial biopsy.
Barge‐Caballero 2021	Retrospective cohort study; Spain; 1998 to 2018.	105	74	75.2%	Patients were diagnosed with cardiac amyloidosis.
Barge‐Caballero 2022	Prospective cohort study; Spain; 2018 to 2020.	128	81	78.1%	Patients in the AMI‐GAL registry with cardiac amyloidosis.
Briasoulis 2022	Retrospective cohort study; Greece; 2015 to 2020.	53	Median 64	57.0%	Patients were diagnosed with AL cardiac amyloidosis.
Cheng 2021	Retrospective cohort study; United States; 2002 to 2019.	309	73	84.1%	Patients were adults with wild‐type or variant ATTR cardiac amyloidosis.
Ioannou 2023	Retrospective cohort study; United Kingdom; 2000 to 2022.	2371	78	90.0%	Patients with ATTR cardiac amyloidosis confirmed at the National Amyloidosis Centre.
Pocari 2021	Prospective cohort study; Italy; 1990 to 2020.	143	‐	‐	Patients with cardiac amyloidosis in the Cardiac Amyloidosis Registry of Trieste.
Ramsell 2022	Retrospective cohort study; United States; 2008 to 2020.	135	72	74.8%	Patients with ATTR or AL cardiac amyloidosis.
Rocha 2022	Prospective cohort study; Portugal; 2019 to 2021.	60	83	80%	Patients with heart failure due to ATTR cardiac amyloidosis.
Tini 2021	Retrospective cohort study; Italy; 2016 to 2020.	642	Median 77	80.8%	Patients with cardiac amyloidosis.
Wanna 2020	Retrospective cohort study; Unclear; 2011 to 2020.	43	Median 65	59%	Patients with AL cardiac amyloidosis.
Yan 2023	Retrospective cohort study; United States; 2012 to 2022.	82	72	82.9%	Patients were adults with heart failure with reduced or mid‐range ejection fraction with a diagnosis of ATTR or AL cardiac amyloidosis.

AL, amyloid light chain; ATTR, transthyretin.

### Demographics and co‐morbidities

The mean age was 74.8 ± 5.5 years across eight studies with available data, and from the 11 studies with data on gender, the proportion of male patients was 75.8%. *Table* *2* shows data on amyloid typing, prevalence of co‐morbidities, and the indication for beta‐blockers. Among the studies that reported on atrial fibrillation/flutter, hypertension, and coronary artery disease, the prevalence of these was 51.3% (1988/3877), 42.0% (1479/3525), and 17.8% (650/3653), respectively. A total of 2181 out of 4255 (51%) patients were on beta‐blocker therapy at the time of diagnosis of CA.

**Table 2:** Summary of studies included detailing the population, prevalence of co‐morbidities, and proportion on beta‐blockers

Study ID	Population	AF/flutter	Hypertension	CAD	Proportion on beta‐blockers	Results
Aimo 2020	ATTR/AL	36/99 (36.4%)	61/99 (61.6%)	‐	86/99 (86.9%)	At median follow‐up of 7.8 months: beta‐blocker withdrawal 7/86. Beta‐blocker dose reduction 15/86.
Austin 2009	ATTR/AL	8/45 (17.8%)	8/45 (17.8%)	10/45 (22.2%)	15/45 (33.3%)	At median follow‐up of 1.7 years: Overall mortality 18/45. Mortality with beta‐blockers versus none: 4/15 versus 14/30. Mortality risk with lack of beta‐blockers: HR 2.88 95% CI 1.25–6.62, aHR 0.98 95% CI 0.98–5.31.
Barge‐Caballero 2021	ATTR/AL	42/105 (40.0%)	53/105 (50.5%)	14/105 (13.3%)	40/105 (38.1%)	At median follow up of 13.7 months: Overall mortality: 49/105. Mortality risk with beta‐blockers: aHR 0.23, 95% CI 0.09–0.59, *P* = 0.002.
Barge‐Caballero 2022	ATTR	72/128 (56.3%)	87/128 (68.0%)	17/128 (13.3%)	65/128 (50.8%)	At median follow up 520 days: Overall mortality: 21/128. Mortality with beta‐blocker versus none: 6/65 versus 15/63, HR 0.18 95% CI 0.08–0.41. No difference in stroke (*P* = 0.23), acute coronary syndrome (*n* = 0.60), arrhythmia (*P* = 0.59), AF/flutter (*P* = 0.22), pacemaker or ICD (*P* = 0.18), syncope (*P* = 0.08), heart failure hospitalization (*P* = 0.16), cardiovascular hospitalization (*P* = 0.12), and all‐cause hospitalization (*P* = 0.99). Beta‐blocker withdrawal 22/65. Adverse effects as reasons for withdrawal 11/22.
Briasoulis 2022	AL	15/53 (28.3%)	10/53 (18.9%)	13/53 (24.5%)	53/236 (22.4%) with AL amyloidosis and cardiac involvement	Beta‐blocker withdrawal 25/53. Overall mortality: 27/53. At median follow up of 17.7 months: Beta‐blocker intolerance and mortality: aHR 3.9, 95% CI 1.26–11.9.
Cheng 2021	ATTR	Baseline 53/309 (17.2%) Follow up 159/309 (51.5%)	‐	20/309 (6.5%)	154/309 (49.8%)	Beta‐blocker withdrawal 66/154. Reason for stopping: 72.7% worsening HF, 59.1% fatigue, 37.9% hypotension, 22.7% bradycardia, 10.6% worsening conduction disease. Baseline beta‐blocker and mortality: a HR 1.37 95% CI 0.81–2.33, *P* = 0.24. Subgroup LVEF <50%: aHR 1.34 95% CI 0.69–2.70. Subgroup LVEF ≥50%: aHR 1.81 95% CI 0.77–4.29. Time varying model showed similar no difference for baseline beta‐blocker (*P* = 0.28). Beta‐blocker stoppage and mortality: aHR 0.44 95% CI 0.22–0.87. Overall mortality: 85/252.
Ioannou 2023	ATTR	1223/2371 (51.6%)	828/2371 (34.9%)	476/2371 (20.1%)	1313/2371 (55.4%)	Beta‐blocker and mortality: aHR 0.90 95% CI 0.78–1.03, *P* = 0.125 Propensity score matched analysis: Overall: HR 0.89 95% CI 0.77–1.04, *P* = 0.149 LVEF ≤40%: HR 0.61 95% CI 0.45–0.83, *P* = 0.002 LVEF >40%: HR 1.00 95% CI 0.84–1.20, *P* = 0.96 Mortality rate 14.9 95% CI 13.9–15.9 deaths per 100 patient‐years during a median follow‐up was 27.8 months.
Pocari 2021	ATTR/AL	‐	‐	‐	‐	At 36 months follow‐up: Beta‐blocker and mortality aHR 0.50 95% CI 0.31–0.82, *P* = 0.007.
Ramsell 2022	ATTR/AL	‐	‐	‐	56/135 (41.5%)	Prior beta‐blocker use 52/135. No beta‐blocker use 27/135. Reason for beta‐blocker withdrawal: hypotension 62%, bradycardia 12%, fatigue 8%, orthostasis 4%, non‐adherence 2%, other 12%.
Rocha 2022	ATTR	‐	‐	‐	60/60 (100%)	Beta‐blocker were reduced or stopped in 40/60 patients, all of whom improved in NYHA class and/or NT‐proBNP (*>*30% reduction) at 1–3 months. Mortality at 2 years 14/60.
Tini 2021	ATTR/AL	364/642 (56.7%)	374/642 (58.3%)	100/642 (15.6%)	250/642 (38.9%)	Beta‐blocker withdrawal 119/642. Intolerance due to HF with advanced diastolic dysfunction 52/119, bradycardia/119, hypotension 21/119, first degree AV block 9/119, high degree AV block 6/119, other 9/119.
Wanna 2022	AL	51%	‐	‐	Baseline 37/43 (86.0%)	Follow up 3 months 59% on beta‐blockers (25/43). Beta‐blocker withdrawal 12/37 (32.4%).
Yan 2023	ATTR/AL	47/82 (57.3%)	58/82 (70.7%)	‐	At diagnosis 52/82 (63.4%)	At mean of 2.1 years follow‐up: Mortality 11/82, but there was no association with baseline or follow up beta‐blocker use. Beta‐blocker withdrawal 10/82. Compared to patients not on beta‐blocker: there was more hypotension (*P* = 0.17) and less syncope (*P* = 0.21) and lightheadedness (*P* = 0.33).

AL, amyloid light chain; ATTR, transthyretin.

### Mortality

**Figure 2:**
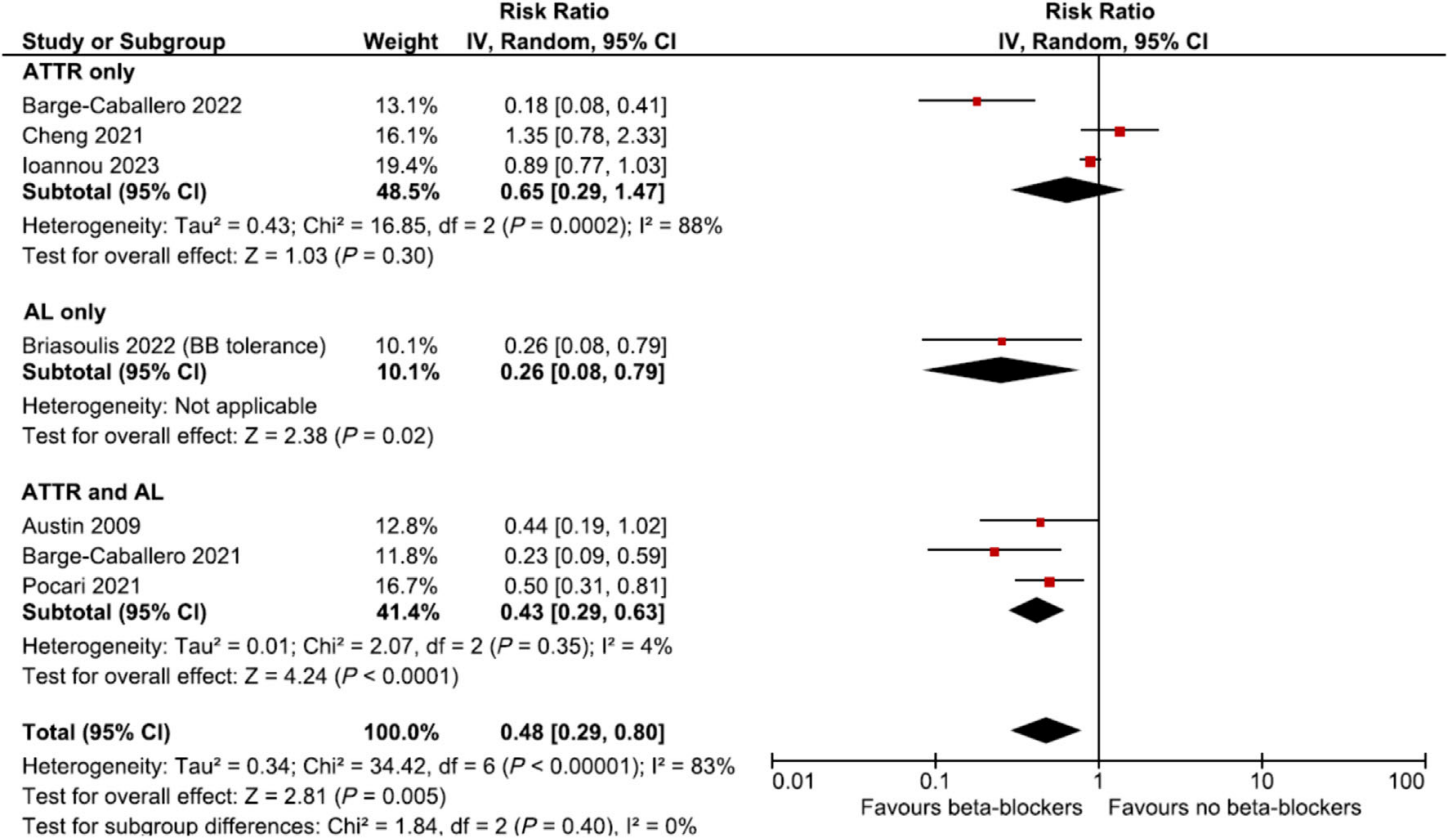
Meta‐analysis assessing risk of all‐cause mortality associated with beta‐blocker use.

Over a median follow up from diagnosis of CA ranging from 13 to 36 months, the pooled mortality was 33.1% (291/879), ranging from 13.4% to 50.9%. There was an inverse association between the pooled risk of mortality and the use of beta‐blocker therapy at any time point (RR 0.48, 95% CI 0.29–0.80, *I* ^2^ = 83%, seven studies) (*Figure* *2*). There was no significant relationship between beta‐blocker therapy and survival in the pooled results of three studies that only included patients with ATTR‐CM (RR 0.65, 95% CI 0.29–1.47, *I* ^2^ = 88%).^8^, ^11^, ^12^ However, there were three studies of populations that included both ATTR‐CM and AL‐CM that suggested a reduction in mortality with beta‐blocker use.^14^, ^22^, ^23^ The study that solely evaluated patients with AL‐CM demonstrated a survival advantage among those patients on beta‐blocker therapy (RR 0.26, 95% CI 0.08–0.79).^15^ Sensitivity analysis identified that exclusion of no single study significantly reduced the statistical heterogeneity. Exclusion of the study by Barge‐Caballero et al.^8^ reduced the *I* ^2^ from 83% to 77%, but the association between reduced mortality and beta‐blocker use remained statistically significant (RR 0.58, 95% CI 0.36–0.92). It is notable that six of the seven studies suggested a directional effect of benefit with beta‐blocker use while the study by Cheng et al. demonstrated a trend towards harm. Exclusion of the Cheng et al. study reduced the overall risk ratio to 0.39 (95% CI 0.21–0.71) but increased the *I* ^2^ = 84%.

### Type and dose of beta‐blocker therapy

The data available on the choice and dosing of beta‐blocker therapy is limited with the exception of the Ioannou et al. study of patients with ATTR‐CM (*n* = 2371).^11^ Of the 1313 patients treated with beta‐blockers, over half were treated with ≤25% of the target dose for heart failure (*n* = 829, 63.1%), and the most commonly prescribed beta‐blocker was bisoprolol (*n* = 1164, 88.7%), with the majority of patients treated with ≤2.5 mg per day (*n* = 721, 61.9%). Only 75 (5.7%) patients had the target beta‐blocker dose prescribed, most of whom had atrial fibrillation (*n* = 58, 77.3%). The overwhelming majority of the study population (*n* = 1266, 96.4%), and all patients with a LVEF ≤40% (*n* = 342, 100.0%) were treated with beta‐blockers.

### Withdrawal of beta‐blocker therapy

Across nine studies, the pooled rate of beta‐blocker withdrawal was 27.4% (353/1287) ranging from 8.1% to 66.7%. Many reasons were given for withdrawal including adverse effects, worsening heart failure, fatigue, hypotension, bradycardia, heart block, worsening condition of the disease, orthostasis, non‐adherence, and advanced diastolic dysfunction.

In the Ioannou et al. study of ATTR‐CM patients, 285 (21.7%) had their beta‐blocker discontinued during follow up [median duration to discontinuation: 14.1 (6.8–28.9) months], and an additional 117 (8.9%) had their beta‐blocker dose reduced [median duration to reduction: 15.7 (7.4–34.5) months]. Patients in whom beta‐blocker therapy was suspended had lower blood pressure and heart rate.^11^

Rocha et al. reported 60 patients with ATTR‐CM among whom discontinuation of beta‐blocker therapy in 40 cases was associated in each case with a subsequent improvement in NYHA class and/or NT‐proBNP (*>*30% reduction) at 1–3 months.^25^ There were no other available studies that assessed the effect of beta‐blocker therapy withdrawal on functional status.

### Cardiovascular outcomes

Barge‐Caballero et al. reported no differences associated with beta‐blocker use in the incidence rates of stroke (*P* = 0.23), acute coronary syndrome (*n* = 0.60), arrhythmia (*P* = 0.59), AF/flutter (*P* = 0.22), pacemaker or ICD (*P* = 0.18), syncope (*P* = 0.08), heart failure hospitalization (*P* = 0.16), cardiovascular hospitalization (*P* = 0.12), and all‐cause hospitalization (*P* = 0.99).^8^

## Discussion

In this study, we evaluated the prescription pattern and discontinuation rates of beta‐blocker therapy in over 4000 patients with CA, and assessed the association between treatment and the risk of mortality. There are several key findings: (i) it remains unclear whether beta‐blockers reduce mortality in patients with ATTR‐CM; (ii) the evidence derived from studies that included both ATTR‐CM and AL‐CM supports an association between beta‐blocker use and improved survival (allowing for one ATTR‐CM study outlier^11^); (iii) more than one in four patients with CA discontinued beta‐blockers, but whether that was because of an inability to tolerate these drugs or because of an active decision to suspend treatment made by clinicians is unclear.

There are three important considerations when examining the observational studies that have assessed the influence of beta‐blockers on survival in patients with CA. First, the indication for beta‐blocker therapy is a confounder and requires close scrutiny. Up to half of patients are in atrial fibrillation or atrial flutter prior to their diagnosis of ATTRwt‐CM. Beta‐blockers may have also been initiated for other common co‐existing conditions including heart failure with reduced ejection fraction, ventricular tachyarrhythmia, myocardial infarction, angina, or hypertension. The perceived survival benefit may therefore relate to the effect of neurohormonal blockade on these conditions rather than direct myocardial effects related to CA. Second, and perhaps more important, is that because the data are taken from observational cohort studies, they are heavily subject to selection and survivor biases. With the exception of the study from Ioannou and colleagues,^10^ there is little or no information available on staging of ATTR‐CM. It is conceivable, therefore, that the apparent survival benefit associated with beta‐blocker therapy reflects the fact that those patients with ATTR‐CM able to tolerate low‐dose beta‐blocker therapy are at an earlier stage of their disease. Patients with early stage disease are known to have higher baseline blood pressures and higher resting heart rates. Third, there may also be publication bias where favourable results are more likely to be reported. Nevertheless, based on the available observational data, the weight of evidence points towards the possibility of survival benefit with beta‐blocker therapy despite a clinical belief that weaning beta‐blockers can improve symptoms.

The lack of high quality evidence relating to conventional heart failure therapy specific to patients with CA has likely contributed to discrepancies between expert consensus guidelines: The ESC recommends discontinuing beta‐blockers in CA regardless of their indication or tolerability,^27^ while the AHA suggests beta‐blockers should still be considered acknowledging that they are often poorly tolerated.^28^ Similarly, the JCS allows treatment with tolerated doses of beta‐blocker in patients with heart failure or for rate control of atrial fibrillation.^29^ Mechanistically, as antagonists of the beta‐adrenergic receptors beta‐blockers deliver negative chronotropic and inotropic effects. While the international heart failure guideline recommendations support beta‐blocker therapy in patients with reduced ejection fraction,^30^, ^31^ the majority of patients with early stage CA present with heart failure with preserved ejection fraction, a population in whom there is no clear evidence of mortality benefit.^32^ While the pathophysiology of CA is such that it typically manifests as a restrictive cardiomyopathy with diastolic dysfunction,^33^, ^34^ in the late stages, it can result in left ventricular systolic dysfunction,^24^ and in ATTRwt‐CM, which is commonly diagnosed in older adults,^35^ patients often have concomitant ischaemic heart disease that can contribute to a reduced ejection fraction. In addition, arrhythmias are common in CA, particularly atrial fibrillation in which beta‐blockers are recommended as first‐line therapy for rate control.^36^

The findings from Cheng et al. contradict those from the remaining beta‐blocker studies that included patients with ATTR‐CM. While this report found no statistically significant association between mortality and beta‐blockade in ATTR‐CM, it was the only study where the direction of the risk estimate pointed towards harm associated with beta‐blockers. This discrepancy could potentially be explained by a low proportion of patients with concomitant coronary artery disease (17.8%) and a higher percentage of missing data (18.4%).

The current observational evidence, while flawed, appears to suggest that beta‐blockers may not need to be routinely suspended in all patients with CA, acknowledging that many patients will be unable to tolerate target dose therapy. This principle may apply to both ATTR‐CM and AL‐CM, although the small proportion of included patients with AL‐CM represents a further limitation of these data. In reality, clinical decision‐making in real‐world practice needs to be individualized and physicians will need to consider the impact of beta‐blocker therapy on quality of life, which in a condition with a poor prognosis, may be more important to patients than prolonging their survival. The median survival for NAC stage 3 disease in ATTR‐CM without TTR directed therapy is only 18 months with mortality higher still for AL‐CM. Therefore, future studies should consider the effect of conventional heart failure therapies on quality of life and functional status as there are limited scientific data focusing on these important outcomes. More evidence is needed, ideally in the form of prospective randomized controlled trials, to definitively determine whether patients with ATTR‐CM and AL‐CM gain prognostic benefit. In such a study, patient selection would be key as choosing patients in the earliest phases of CA will improve tolerability but may not yield many events, while choosing patients with later stage disease will likely impact tolerability, despite yielding higher event rates.

## Limitations

Our findings reflect real‐world global practice encompassing evidence from North America, the United Kingdom, and Europe. Nonetheless, this review has several limitations. An individual patient‐level meta‐analysis would have been the favoured method for pooling studies and outcomes,^37^ but such an approach did not align with our aim of objectively reviewing all studies related to this topic. All data are taken from observational studies and will, therefore, be subject to selection, information, performance, follow‐up, ascertainment, and survivor biases. Without randomization, the deterministic effect of an intervention is influenced by the prescription patterns of clinicians. Many of the included studies are small‐scale and some included patients with both ATTR/AL to achieve a larger sample size. Further, we were seldom able to elicit the indication for beta‐blockers. In addition, we did not have available data on left ventricular ejection fraction and so were unable to assess the impact of beta‐blockers on outcomes according to the presence of reduced versus preserved ejection fraction. Finally, the reporting of the studies did not have the sample size or sufficient granularity in the data to be able to account for the effects of other prescribed medications or ascertain the effect of beta‐blocker therapy on basic physiological measures like blood pressure and heart rate.

## Conclusions

The current findings from observational studies suggest that better quality evidence is needed to ascertain the true effect of beta‐blockers on mortality in patients with CA. The studies evaluating the longitudinal use of beta‐blocker therapy suggest that more than a quarter (27%) of patients treated at baseline had their treatment withdrawn. These observational data are retrospective and subject to serious selection and survivor biases. Prospective randomized trials are therefore required to examine the efficacy and tolerability of beta‐blockers in patients with cardiac amyloidosis with respect to survival, hospitalization for heart failure, quality of life, and functional status. However, recruitment and retention into such trials will likely be challenging due to the poor tolerability of beta‐blockers in late stage CA.

## Conflict of interest

WEM has received speaker/advisory board fees from Pfizer, Alnylam, Akcea, Sobi, BMS, and Boehringer‐Ingelheim.

## Funding

None.

## Supporting information


**Data S1.** Supporting Information.

**Table S1.** Study quality assessment.

## Data Availability

The data were derived from published literature, and requests for further data need to be made from the authors of the original studies.
