# Supplementary material for: Effect of beta‐blockade on mortality in patients with cardiac amyloidosis: A systematic review and meta‐analysis
Source: ESC Heart Fail. 2024 Jul 23;11(6):3901–10. doi: 10.1002/ehf2.14975 (PMC11631279; doi:10.1002/ehf2.14975)
Supplement: Supplementary file 2 — Table S1. Study quality assessment. [file EHF2-11-3901-s001.docx]

**Supplementary Table 1. Study quality assessment**

| **Study ID** | **Representativeness of cohort (Is the cohort ATTR only?)** | **Selection of non-exposed cohort (Is the group without β-blockers reliable?)** | **Ascertainment of exposure (Is β-blockers exposure reliable?)** | **Demonstration of outcomes not present at start of study (Death or safety)** | **Comparability (Are the results adjusted?)** | **Assessment of outcome (Is the outcome assessment reliable?)** | **Follow up adequacy (Was there in-hospital outcomes or follow up?** | **Low missing data (<10%)** | **No. of stars out of 9** |
| --- | --- | --- | --- | --- | --- | --- | --- | --- | --- |
| Aimo 2020 | No, ATTR/AL | Yes | Yes | Yes, safety | No, unadjusted | Yes, safety | Yes, median 7.8 months | Yes, not reported | 6 |
| Austin 2009 | No, ATTR/AL | Yes | Yes | Yes, mortality | Yes, adjusted | Yes, mortality | Yes, median 1.7 years | Yes, not reported | 8 |
| Barge-Caballero 2021 | No, ATTR/AL | Yes | Yes | Yes, mortality | Yes, adjusted | Yes, mortality | Yes, median 13.7 months | Yes, not reported | 9 |
| Barge-Caballero 2022 | Yes, ATTR only | Yes | Yes | Yes, mortality | Yes, inverse probability weighted | Yes, mortality | Yes, median 520 days | Yes, 9/128 missing data | 9 |
| Briasoulis 2022 | No, AL only | Yes | Yes | Yes, mortality | Yes, adjusted | Yes, mortality | Yes, median 17.7 months | Yes, not reported | 8 |
| Cheng 2021 | Yes, ATTR only | Yes | Yes | Yes, mortality | Yes, adjusted | Yes, mortality | Yes, data locked in 2019 when recruitment ended 2018 | No, 57/309 missing data | 8 |
| Ioannou 2023 | Yes, ATTRwt-CM only | Yes | Yes | Yes, mortality | Yes, adjusted | Yes, mortality | Yes, median follow up 27.8 months | Yes, 202/2371 missing data | 9 |
| Pocari 2021 | No, ATTR/AL | Yes | Yes | Yes, mortality | Yes, adjusted | Yes, mortality | Yes, 36 months | Yes, not reported | 8 |
| Ramsell 2022 | No, ATTR/AL | Yes | Yes | Yes, safety | No, unadjusted | Yes, safety | Not reported | Yes, not reported | 5 |
| Rocha 2022 | Yes, ATTR only | Yes | Yes | Yes, safety | No, unadjusted | Yes, safety | Yes, 2-year follow up. | Yes, not reported | 7 |
| Tini 2021 | No, ATTR/AL | Yes | Yes | Yes, safety | No, unadjusted | Yes, safety | Yes, median follow up 419 days | Yes, not reported | 7 |
| Wanna 2022 | No AL only | Yes | Yes | Yes, safety | No, unadjusted | Yes, tolerance. | Yes, 3 months. | Yes, not reported | 6 |
| Yan 2023 | No, ATTR/AL | Yes | Yes | Yes, safety and mortality. | No, unadjusted | Yes, safety and mortality. | Yes, mean 25 months | Yes, not reported | 6 |

ATTR=transthyretin; AL=amyloid light chain
